# Optimal Technique for Introducing Schwann Cells Into Peripheral Nerve Repair Sites

**DOI:** 10.3389/fncel.2022.929494

**Published:** 2022-07-01

**Authors:** Emily L. Errante, Anthony Diaz, Taylor Smartz, Aisha Khan, Risset Silvera, Adriana E. Brooks, Yee-Shuan Lee, S. Shelby Burks, Allan D. Levi

**Affiliations:** ^1^The Miami Project to Cure Paralysis, University of Miami Miller School of Medicine, Miami, FL, United States; ^2^Department of Neurological Surgery, University of Miami Miller School of Medicine, Miami, FL, United States; ^3^Interdisciplinary Stem Cell Institute, University of Miami Miller School of Medicine, Miami, FL, United States

**Keywords:** peripheral nerve, nerve injury treatment, regeneration, Schwann cells, guidance channel

## Abstract

Peripheral nerve injury (PNI) is found in a relatively large portion of trauma patients. If the injury is severe, such as with the presence of a long segmental gap, PNI can present a challenge for treatment. The current clinical standard of nerve harvest for the repair of long segmental gap PNI can lead to many potential complications. While other methods have been utilized, recent evidence indicates the relevance of cell therapies, particularly through the use of Schwann cells, for the treatment of PNI. Schwann cells (SCs) are integral in the regeneration and restoration of function following PNI. SCs are able to dedifferentiate and proliferate, remove myelin and axonal debris, and are supportive in axonal regeneration. Our laboratory has demonstrated that SCs are effective in the treatment of severe PNI when axon guidance channels are utilized. However, in order for this treatment to be effective, optimal techniques for cellular placement must be used. Thus, here we provide relevant background information, preclinical, and clinical evidence for our method in the treatment of severe PNI through the use of SCs and axon guidance channels.

## Introduction

Peripheral nerve injury (PNI) occurs in approximately 3% of all trauma patients (Nadi et al., 2018). It is characterized by a loss of cellular and axonal integrity that is replaced by scar and neuromatous tissue, producing deficits in sensory/motor function, and, not infrequently, an associated neuropathic pain syndrome. While the severity and complexity of the injury may vary, many PNIs are not currently amenable to repair with traditional techniques of sural nerve grafting. While nerve transfers have demonstrated success in brachial plexus injuries, many lower extremity nerve injuries have no nerve transfer options. For such injuries, traditional nerve grafting has seen limited success in the clinical setting. The absence of a therapy to substantially ameliorate the detrimental effects of PNI has led to numerous chronically paralyzed individuals all over the world (Wilcox et al., 2021).

Cell therapies, particularly those that utilize Schwann cells (SCs), offer the promise of being able to provide a means of neural tissue and functional replacement; they can provide a broad range of beneficial actions due to the intricate complexities and functionality of a cell compared to a single biological or pharmacological agent. However, it is imperative that an optimal technique is used to introduce cell therapies into a nerve injury site. Through extensive preclinical and clinical assessment over the last several years (Burks et al., 2014, 2021; Gersey et al., 2017), our laboratory has demonstrated success in both humans and rodents through the use of specific methods that we believe to be ideal for PNI, particularly those injuries that have a large nerve gap defect.

## Peripheral Nerve Injury: Repair With Significant Segmental Defects

Repair of nerve injury, particularly when a significant gap exists, represents one of the most difficult challenges in peripheral nerve surgery. Insufficient autologous donor material remains one of the major obstacles in obtaining functional motor and sensory recovery (Kim et al., 2004; Gousheh et al., 2008; Murovic, 2009; Aydin et al., 2010). Repair of sciatic transection with the use of nerve autograft has yielded variable success in the past and, from these studies, we see inconsistent outcomes depending on the level of injury and whether the tibial or peroneal branches were involved (Kim et al., 2004; Gousheh et al., 2008; Aydin et al., 2010). Focusing on repair requiring autologous nerve graft, the worst outcomes are seen in the peroneal division of the sciatic nerve at the level of the buttock (Kim et al., 2004; Gousheh et al., 2008). In contrast, somewhat higher rates of recovery are seen with tibial graft repairs in the mid-thigh. In addition to the location of injury within the leg and branch involved, other factors cited to affect the success of nerve grafting include the length of time to surgery (Grinsell and Keating, 2014) and a nerve defect or gap >5 cm (Pan et al., 2020), both of which are associated with worse outcomes.

The sural nerve is a common candidate for autologous nerve grafting as it represents a non-critical sensory nerve leaving a relatively small area of sensory deficit after harvest (Riedl et al., 2008; de Alvarenga Yoshida et al., 2012; Kerver et al., 2012; Riedl and Frey, 2013). Cadaveric studies have demonstrated that the maximum obtainable average sural nerve length is approximately 43 cm per leg with a range of 35–47 cm (Riedl et al., 2008). In cases of lengthy traumatic sciatic nerve defects, it is beneficial to know, given the relatively small diameter of the sural nerve, what length of harvestable sural nerve would be required to repair a given defect of the sciatic nerve. A critical factor in the determination of the maximum length of the repairable sciatic nerve is the cross-sectional area of both the sural and sciatic nerves in any given patient. We studied sural and sciatic nerve samples from human cadavers to evaluate the cross-sectional area (CSA) of each of these nerves and determined that numerous sural nerve grafts must be cut, layered, and sutured across a human sciatic nerve gap to bridge a traumatic defect (Higgins et al., 2002; Gousheh et al., 2008; Karabekmez et al., 2009; Hobson-Webb et al., 2013; Burks et al., 2014). We determined that there was an incredible variability in the CSA of individual sural nerve grafts ranging from a low of 1.20 mm^2^ to a high of 3.75 mm^2^. Using these measurements, we estimate, using a 95% confidence interval, that patients with sural nerves exhibiting a small CSA may only be able to cover a 2.5 cm sciatic nerve defect whereas those with larger CSA may bridge up to an 8 cm gap (Burks et al., 2014). Insufficient autologous nerve is a major obstacle to successful repair strategies for sciatic nerve injuries when the gap exceeds lengths of 6 cm, indicating a need for different techniques for these types of PNI.

## Use of Axon Guidance Channels

In cases of peripheral nerve defects with long gaps, artificial axon guidance channels (AGC) can promote nerve regeneration and prevent neuroma formation (Tyner et al., 2007). It has been shown that each animal species has its own upper limit for the maximum nerve gap distance that can be effectively repaired by this type of empty acellular conduit (Lundborg et al., 1982a, b). In the rat sciatic nerve, this critical distance measures approximately 13 mm, and in the primate ulnar nerve, it is 3 cm (Williams et al., 1987; Hess et al., 2007). Nerve injuries with long segmental defects in humans pose a similar challenge in which gap lengths of more than 6 cm have extremely poor clinical outcomes (Pan et al., 2020). Research in primates supports the clinical use of NeuraGen (Integra Lifesciences Corp.) tubes for gaps up to 30 mm; thus, these tubes are commonly used to repair gaps <3 cm in humans (di Summa et al., 2014).

Recently, preclinical data using second-generation ACGs for nerve repair offers promise compared to autologous nerve grafting (Lee et al., 2012). These ACGs represent the perfect platform for the delivery of growth-enhancing substrates. Such substrates studied in the past include nerve growth factor (NGF), brain-derived neurotrophic factor (BDNF), glial growth factor (GGF), denatured muscle, small segments of peripheral nerve, and, recently in our own labs, purified Schwann cells (SCs; Berrocal et al., 2013). The addition of SCs to AGCs has demonstrated enhanced axonal regeneration and improved functional recovery in mice, rats, and nonhuman primates (Levi et al., 1994, 1997; Berrocal et al., 2013). SCs are able to promote axonal growth, in part, through the release of neurotrophic factors (Richardson et al., 1982; Ide et al., 1983; Kim et al., 1994; Bryan et al., 1996). This ability makes SCs an excellent supplement to acellular guidance channels.

## Human Schwann Cells Are Able to Retain Their Functional Capability

SCs are an integral part of peripheral nerve regeneration and restoration of function (Jessen and Mirsky, 2019; Nocera and Jacob, 2020; Stassart and Woodhoo, 2021). After an injury, axonal degeneration occurs distal to the injury site in a process called Wallerian degeneration (Gaudet et al., 2011). While severed peripheral nerves remain intact for several days after injury, they ultimately degenerate in both rodents and humans (Chaudhry and Cornblath, 1992; Beirowski et al., 2005). After PNI, SCs de-differentiate to a proliferating/repair state (Mirsky et al., 2008; Jessen and Mirsky, 2019). Among other things, SCs downregulate myelin production (Jessen and Mirsky, 2016; Stratton et al., 2018), which is essential for the creation of an environment for axons to regrow (Hirata and Kawabuchi, 2002; Stratton et al., 2018). Once myelin debris is cleared, axonal regrowth can occur. Together with other molecules, SCs are able to promote axonal repair and regeneration. Due to their role in the normal repair response, transplanted SCs are able to support nerve repair after PNI.

Researchers at the Miami Project to Cure Paralysis over the last quarter-century have been responsible for developing an efficient method for procuring large, essentially pure populations of human SCs from adult peripheral nerves. Investigations to define culture methods were initially performed with adult rat SCs and then extended to other species, including primates and ultimately human sources (Morrissey et al., 1991). Expanded adult-derived rat SCs retain their functional capacity as evidenced by their ability to myelinate dorsal root ganglion neurites and to support the regeneration of processes from embryonic rat retinal explants *in vitro* (Morrissey et al., 1991). SCs also functioned *in vivo* when SC-seeded guidance channels placed in an 8 mm gap of a transected sciatic nerve supported extensive peripheral nerve regeneration (Guenard et al., 1992; Levi and Bunge, 1994; Levi et al., 1994).

In addition to determining the most efficient method for the cultivation of adult human SCs, the Miami Project group has evaluated the functionality of the expanded cells. The first evidence that human SCs could ensheathe either rat or human DRG axons was seen in tissue culture (Morrissey et al., 1995) and in *in vivo* studies where human SCs were transplanted into immune-deficient rodents with sciatic nerve transection (Levi and Bunge, 1994; Levi et al., 1994) or thoracic spinal cord transection (Guest et al., 1997a, b). These studies showed that human SCs that have been expanded in culture with mitogens can survive and are capable of enhancing axonal regeneration and forming myelin after transplantation. The reproducibility of the technique for isolating human SCs from peripheral nerve also was confirmed in a study that evaluated the purity of human SC preparations from donors aged 1 to 63 years. The average SC purity from all donors was 92.7 ± 2.73%. The age of the donor and the length of explant culture time did not appear to influence the purity of human SC preparations (Levi, 1996). More recent work in humans has also indicated purity above 90% and has shown that longer nerve harvest lengths are correlated with higher yields of SCs in culture (Khan et al., 2021). When combined with the extensive preclinical data submitted for FDA approval of autologous human SCs for subacute and chronic spinal cord injury (SCI), the record for safety of autologous human SC transplantation is extensive (Anderson et al., 2017; Khan et al., 2021; Gant et al., 2022).

## Rodent Model of PNI: Technique and Results

Due to several limitations in current treatment methods and the clear benefits of SCs in PNI, particularly for long segmental gap PNI, it was essential to develop an optimal treatment method for SC delivery into injury sites. In the first study to examine this (Burks et al., 2021), NeuraGen 3D nerve guides were used (Integra Lifesciences Corp., New Jersey, USA). This product is manufactured from a highly purified type 1 collagen derived from the bovine deep flexor tendon. The NeuraGen 3D nerve guides were specifically chosen due to the fact that they contain an internal lattice structure that replicates the longitudinal endoneurial tubes, which have been shown to be more efficacious compared to hollow conduits (Lee et al., 2012). The nerve guides used in the study had an internal diameter of 1.5 mm and a length of 15 mm.

**Figure 1:**
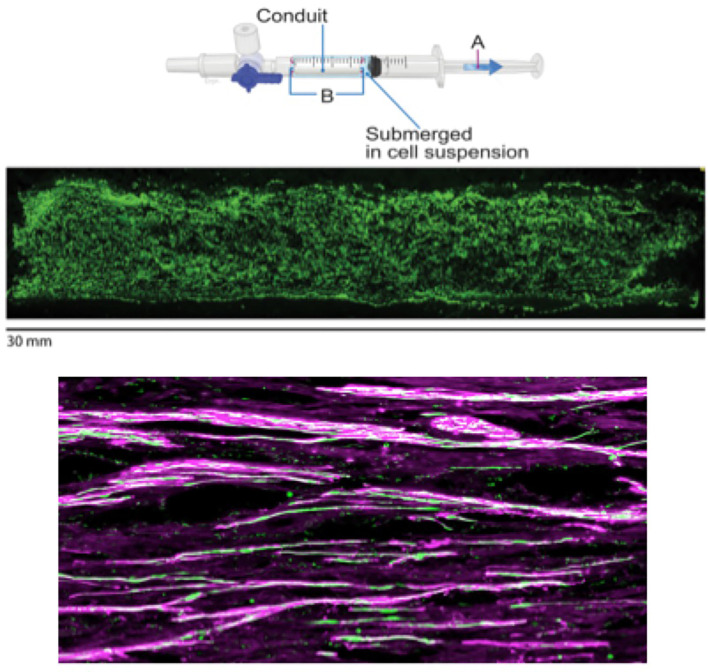
Figure showing SC loading, SC distribution within the conduit, and the presence of SCs within regenerated nerve fibers. Top two figures borrowed from Burks et al. (2021) demonstrating SC loading of NeuraGen 3D conduits. Loading of SCs into the conduit (upper panel) was done by first submerging the dry 15-mm conduit **(B)** inside of a 1-ml syringe (for animal model) filled with DMEM-suspended SCs at a concentration of 100,000 cells/μl and then applying negative pressure with a syringe plunger **(A)**. A homogenous distribution of GFP-labeled SCs (middle panel) can be observed with this method of loading. For visualization purposes, a longitudinal section of a human-sized conduit (30 mm) loaded (using 2.5 ml of solution in a 3-ml syringe) with GFP-labeled SCs is included. The bottom figure shows the presence of GFP-labeled SCs (green) within the regenerated nerve fibers (purple), helping to show the efficacy of the treatment. Upper panel copyright Roberto Suazo. Published with permission.

A 13-mm segment of the sciatic nerve was removed *via* sharp transection in all animals in the study. While other treatments were examined in the study for comparison purposes, one group received the NeuraGen 3D nerve guide filled with green fluorescent protein (GFP)-labeled SCs. The loading step took place 2–4 h before surgery. This loading technique involves suspending SCs into a DMEM solution at a concentration of 100,000 cells/μl. A total volume of 350 μl was placed into a 1-ml syringe, as this was the lowest volume of cell solution that would completely submerge the NeuraGen 3D nerve guide. The dry 15-mm conduit was then placed into the syringe and, through the application of negative pressure to the chamber with the syringe plunger, the cells are driven into the dry conduits (Figure 1; Burks et al., 2021).

Results from the study indicated that the loading method was the optimal technique for cellular delivery to a PNI site. Specifically, the SCs were confirmed to have survived, as indicated by the presence of GFP-labeled SCs that were seen within the regenerated nerve fibers (Figure 1). It was also shown that, with this method, regeneration and elongation of myelinated axons in all segments of the graft were significantly enhanced by the end of the study (16 weeks) in the SC-filled Neuragen 3D nerve guide when compared to animals that had received an empty nerve guide. Further, these findings were similar to animals that had received reversed autograft, the “gold standard” of treatment for PNI. Additionally, nerves that were repaired with the SC-filled nerve guides demonstrated electrophysiological results similar to control animals and had significantly less muscle atrophy compared to the group that was treated with an empty conduit (Burks et al., 2021).

Importantly with this loading method, there is a homogenous distribution of SCs within the conduit (Figure 1). This has been further shown in the lab through testing and visualization of various concentrations of SCs within the Neuragen 3D nerve guides (unpublished data). This is an important consideration when discussing the optimal technique for introducing SCs into PNI sites, as uniform cellular distribution within an injury site is desirable. Specifically, this was seen in the current study through the interaction of SCs with regenerating axons throughout all segments of the Neuragen 3D nerve guide, which was measured at various time points throughout the study. This suggests that in severe PNI, exogenously implanted SCs are capable of long-term survival and continue to play a functional role in axonal regeneration (Burks et al., 2021). Taken together, these findings provide clear support for an optimal technique for the introduction of SCs into a PNI site in animal models, which helped to support recovery in all animals given the treatment.

## Human Cases of PNI: Technique and Results

While the laboratory has had great success in rodent models of severe PNI, this success has also been translated into clinical practice. In this translational process, we must first demonstrate the benefit of adding cells to the traditional nerve repair, i.e., suralautograft. The first patient was enrolled in 2014 and several have since been enrolled with long-term follow up (Gersey et al., 2017). These are patients with long-segment, severe peripheral nerve injuries.

In the initial two cases, both patients experienced significant trauma to the lower extremity. Specifically, Patient 1 sustained the injury after a boat propeller accident while Patient 2 had a gunshot wound. Both injuries were explored initially as was deemed appropriate and a small segment of the sural nerve was harvested to facilitate SC culture for each patient. Sciatic nerve repair and SC transplantation took place within 3 weeks after SC harvest in both patients. This 3-week gap allowed the proximal and distal nerve stumps to define themselves, and is standard in blunt transection type injuries. SCs were obtained from both the sural nerve and from the damaged sciatic nerve stump. They were then expanded and purified in culture (Gersey et al., 2017). Briefly, this was done by placing passage 0 cells into flasks coated with mouse laminin and feeding them with a culture medium every 3 days until they reached 80%–90% confluence after 7 days (Khan et al., 2021). Once the cells reached confluence, the flasks were rinsed with Hanks’ Balanced Salt Solution (Thermo Fisher Scientific, Massachusetts, USA) and incubated in TrypLE Select (Thermo Fisher Scientific, Massachusetts, USA; Khan et al., 2021). D10 medium was then added to each flask and cell suspensions were collected, centrifuged, and passage 1 cells were then resuspended with D10 medium (Khan et al., 2021). Viability, total cell count, and purity were all determined at this stage (Khan et al., 2021).

**Figure 2:**
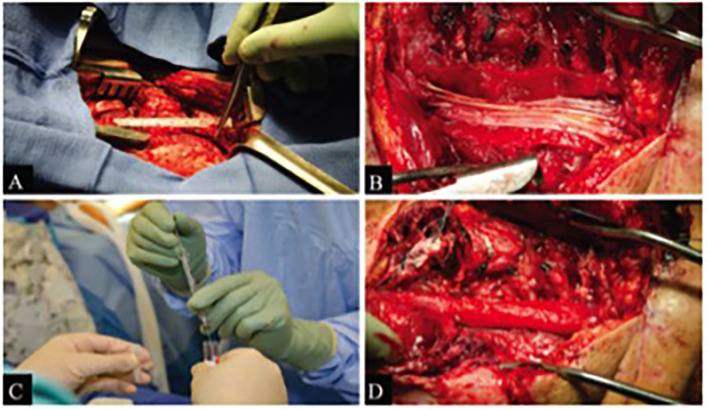
Figure borrowed from Gersey et al. (2017) demonstrating intraoperative views of sciatic nerve repair with SC transplantation in Patient 1. **(A)** Initial exposure of nerve damage. **(B)** Sural nerve repair with Duragen graft in background. **(C)** Nerve construct supplemented by SCs. **(D)** Duragen graft sewn around nerve/SC construct.

In the case of the patient that was in a boat propeller accident (Patient 1), the sciatic nerve was completely transected with a measured defect of 7.5 cm. Sural nerves were harvested, placed, and sutured. Approximately 28 million SCs were supplemented within a Duragen Secure Dural Regeneration Matrix (Duragen; Integra Lifesciences Corp., New Jersey, USA), which was then sewn around the nerve/SC construct (Figure 2). In the case of the patient with the gunshot wound (Patient 2), only the tibial component of the nerve was damaged, with the defect measuring 5 cm. After the sural nerve was obtained, the nerve grafts were placed and sutured. 110 million autologous SCs were supplemented within a Duragen Secure Dural Regeneration Matrix, which was sewn around the nerve/SC construct similar to Patient 1 (Gersey et al., 2017). This collagen sheet provides a physical boundary for the transplanted cells. The construct is further secured with the use of a fibrin glue, which also keeps cells in close physical proximity to the regenerating axons.

Long-term follow-up with each patient indicated the procedures were successful. Specifically, in Patient 1, motor function was regained in the tibial distribution, with partial function in the peroneal distribution. The patient also had a partial return of sensory function with complete resolution of neuropathic pain. Patient 2 regained complete motor function with partial return of sensation. Postoperative imaging also supported the recovery in both patients, with MRI and ultrasound demonstrating nerve graft continuity and no tumor formation at the repair site (Gersey et al., 2017).

Overall, these findings demonstrate an optimal method for introducing SCs into severe PNI sites in clinical practice. This is indicated by the significant improvements seen in both patients in sensory and motor function. Importantly, the study also indicates the safety and efficacy of SC transplantation for peripheral nerve repair. This was the first study to present successful cases through the use of these methods. Considering the drawbacks of historically used treatment methods compared to the efficacy of the treatment approach used here, it seems that the described methods for clinical treatment are promising.

## Discussion

PNI is a commonly seen injury in trauma patients; however, severe PNI presents a challenge to surgeons due to several limitations of previously available methods, including lack of donor tissue and possible formation of neuroma. Due to the drawbacks of these methods, researchers have looked at different techniques, including the introduction of SCs into the injury site. While it is widely recognized that SCs are beneficial to the injury site, it is essential that the optimal technique be utilized to introduce SCs into the repair site. Our laboratory has established both preclinical- and clinically-based techniques for the introduction of SCs into large segmental gap PNIs with much success. Specifically, through the introduction of purified SCs into constructs like axon guidance channels with a specific internal structure, the SCs are able to retain their functionality long-term to assist in nerve regeneration at the injury site. Importantly in both rodent models and in clinical trials, significant recovery is observed. Thus, the previously described methods represent optimal techniques for the introduction of SCs into PNI sites.

## Data Availability Statement

The original contributions presented in the study are included in the article, further inquiries can be directed to the corresponding author.

## Ethics Statement

The studies involving human participants were reviewed and approved by FDA and University of Miami Miller School of Medicine Institutional Review Board. The patients/participants provided their written informed consent to participate in this study. The animal study was reviewed and approved by IACUC University of Miami Miller School of Medicine. Written informed consent was obtained from the individual(s) for the publication of any potentially identifiable images or data included in this article.

## Author Contributions

All authors contributed to the article and approved the submitted version.

## Conflict of Interest

The authors declare that the research was conducted in the absence of any commercial or financial relationships that could be construed as a potential conflict of interest.

## Publisher’s Note

All claims expressed in this article are solely those of the authors and do not necessarily represent those of their affiliated organizations, or those of the publisher, the editors and the reviewers. Any product that may be evaluated in this article, or claim that may be made by its manufacturer, is not guaranteed or endorsed by the publisher.
